# An Address, Delivered before the Suffolk District Medical Society, at Its Second Anniversary Meeting, Boston, March, 1851

**Published:** 1851-05

**Authors:** 


ART. XI.—*An Address*, delivered before the Suffolk District Medical Society, at its Second Anniversary Meeting, Boston, March, 1851. By SAM'L PARKMAN, M. D., M. M. S. S., one of the Surgeons of the Massachusetts General Hospital.

This is a philosophic view of medicine, but contains some concessions that we are not prepared to admit. The following remarks seem to us to be untrue to that spirit of investigation that has been at work for the last eighteen years, among medical men. Facts do not bear out the charge that "medicine has remained inefficient and powerless, as regards the dscovery of the real cause and nature" of cholera. Dr. Parkman pleads guilty to the charge, and justifies himself in the following language:

Medicine has been reproached because, in the presence of that scourge, the cholera, which has, with mysterious and deadly step, passed over nearly the whole world, it has remained inefficient and powerless as regards the discovery of its real cause and nature; not even having pointed out a sign by which its approach might be foreseen, even if it were ordained that we must succumb to its devastation, as to a decree of fate. The charge is true; but let only those who are free in this respect, cast the reproach. Has any naturalist ever approximated to the cause or nature of the disease, which in like manner has spread over a large portion of the world, depriving a nation of its subsistence? And yet, which of the two has the most advantages and facilities for arrival at discovery, the one compelled to judge of the hidden processes of disease by obscure signs, or only able to examine its effects after its ravages have been completed; or the other, who has merely to walk into a potato field and make living autopsies of his diseased patients? A mysterious influence has traversed the land, and a class of forest trees, which had for years with each returning spring spread their leaves for grateful shade, now raise their bare branches—a deformity and a barrenness in the midst of the beauty and plenteousness of summer. Has any one shown the causes of this? Has any one advanced even a tenable theory, or made so much as a probable supposition?

We are confident that the cause, nature and phenomena of cholera are as perfectly understood as those of any other disease. A great variety of theories and whims has been started on the subject, as there is in every prominent matter of human interest, but surely medical men may sift fact from falsehood, and know when they have the truth. Volney took a lofty attitude, in his estimation, in philosophy, and looked down contemptuously on the struggles of the various religions of the earth; and after surveying them narrowly, as he thought, he announced

"that here was a variety of religions, all claiming a divine origin, but inasmuch as no one of them would tolerate the claim of any but its own, therefore all were false." He would not investigate; he would not go through a patient analysis of evidence upon the highest question ever submitted to man, but skimmed over the surface of things and toppled over into the mire we have recorded. And thus it is in the investigation of epidemic influences. They demand a great variety of knowledge, on a great variety of subjects, a thorough and extended mastery of all the facts, patient and laborious analyses, and rather than undertake the acquisition and use of these powers, men will listen to the clamors of conflicting theories, and sagely conclude there is no truth, or if there is, it is unknown. These are not the methods by which scientific medicine should be advanced.

How can Dr. Parkman make such an admission as he does in the above extract from his address, in the presence of such labors as those that have characterized the sanitary survey of Great Britain? Have not Chadwick, Grainger, Guy, and a host of other British minds, and Riecke, of Prussia, thrown some light on "the real cause and nature of cholera?" Pray tell us, what disease can medical men honestly claim a better acquaintance with, than with cholera?

With the exception noted, we are much pleased with the spirit and style of Dr. Parkman's address. B.

